# Association of medication regimen complexity index with ADRs in HIV/AIDS patients: a retrospective cohort study

**DOI:** 10.3389/fphar.2025.1721289

**Published:** 2026-01-12

**Authors:** Ji Sun, Hui Qi, Jingmeng Huang, Ying Wang, Yu Xiang, Gefei He, Shiqiong Huang

**Affiliations:** 1 The Affiliated Changsha Hospital of Xiangya School of Medicine, Central South University, Changsha, Hunan, China; 2 Department of Pharmacy, The first hospital of Changsha, Changsha, Hunan, China; 3 Department of Infection and Immunology, The first hospital of Changsha, Changsha, Hunan, China; 4 The First People’s Hospital of Zixing, Chenzhou, Hunan, China; 5 Changsha County Hospital of Traditional Chinese Medicine, Changsha, Hunan, China; 6 People’s Hospital of Baojing County, Xiangxi Tujiaand MiaoAutonomous Prefecture, Hunan, China

**Keywords:** adverse drug reactions, aids, HIV, Mrci, real-world

## Abstract

**Purpose:**

To investigate the correlation between the medication regimen complexity index (MRCI) and adverse drug reactions (ADRs) among acquired immunodeficiency syndrome (AIDS) patients in the Chinese population.

**Introduction:**

The complexity of antiretroviral therapy (ART) regimens in individuals living with human immunodeficiency virus (HIV) presents significant challenges to medication management. To date, no studies have investigated the correlation between the MRCI and ADRs.

**Methods:**

This study retrospectively enrolled 1,010 patients. The MRCI was utilized to quantify the complexity of pharmacological treatment regimens. All suspected ADRs were assessed for causality using the World Health Organization-Uppsala Monitoring Centre (WHO-UMC) system. Univariate and multivariate logistic regression analyses were conducted to identify risk factors associated with ADRs and MRCI.

**Results:**

Our findings demonstrate that the MRCI significantly increased from 4.09 ± 4.04 pre-admission to 6.09 ± 6.05 at discharge (*P* < 0.0001). Comorbidities (>6 diseases) (OR: 2.85, *P* < 0.001), tuberculosis medications (OR: 1.82, P < 0.001), and the number of medications administered during hospitalization (>19 drugs) (OR: 2.02, *P* < 0.001) were identified as independent factors influencing MRCI levels. The AUC for predicting adverse reactions using MRCI was 0.58 (*P* = 0.001), with an optimal cutoff value of 20.75. MRCI (>20.75) (OR: 1.42, *P* = 0.036) emerged as independent risk factors for adverse reactions in HIV/AIDS patients.

**Conclusion:**

In China, the MRCI of HIV/AIDS patients significantly increased after hospitalization. Further analysis indicates that patients with higher MRCI are more likely to experience ADRs.

## Introduction

The advent of highly active antiretroviral therapy (HAART) has transformed HIV infection into a chronic manageable condition, enabling people living with Human Immunodeficiency Virus (HIV) to achieve near-normal life expectancy (Deeks et al., 2013; Poorolajal et al., 2016). HAART creates a potent attack by combining three or more drugs with different mechanisms, thereby maximally suppressing the HIV virus and preventing drug resistance (Lu et al., 2018). However, it inevitably entails the complexity of multiple - drug regimens. Particularly among elderly patients, who often present with age - related complications, this leads to complex polypharmacy regimens and an increased risk of drug interactions (Abdelbary et al., 2023; Back and Marzolini, 2020). The complexity of polypharmacy significantly elevates the incidence of adverse drug reactions (ADRs) (Yan et al., 2025). Existing research demonstrates that ADRs are prevalent among HIV-infected individuals undergoing antiretroviral therapy (ART) (Sherfa et al., 2021).

The complexity of medication regimens reflects the multidimensional nature of prescriptions, encompassing not only the quantity of drug classes but also various aspects of prescription practices. Currently, the Medication Regimen Complexity Index (MRCI) stands as the most widely utilized quantitative tool for assessing regimen complexity (George et al., 2004). This instrument has been translated and validated in multiple languages, including German (Stange et al., 2012), Spanish (Saez de la Fuente et al., 2016), Portuguese (Melchiors et al., 2007), Turkish (Okuyan et al., 2016), Korean (Lee et al., 2019), Japanese (Masumoto et al., 2021) and Chinese.

The MRCI influences patients’ risk of readmission, medication adherence, potential inappropriate medication use, quality of life, and other factors. (Abdelbary et al., 2023; González-Bueno et al., 2022; He et al., 2023; Sayın et al., 2022; Wilkening et al., 2020). Current research on MRCI values in patients with chronic conditions includes studies on chronic obstructive pulmonary disease (He et al., 2023), chronic heart failure (Wilkening et al., 2020) and acquired immunodeficiency syndrome (AIDS) (García et al., 2025). One study identified an MRCI threshold of 11.25 for HIV/AIDS patients meeting polypharmacy criteria (Morillo-Verdugo et al., 2019). Higher MRCI indices correlate with poorer quality of life in HIV-infected patients (Contreras-Macías et al., 2021). Furthermore, research demonstrates that elevated baseline MRCI are significantly associated with hospitalizations due to ADRs, with risk markedly increasing when MRCI exceed eight points (Pooja et al., 2024).

However, there is currently no published research investigating the association between the MRCI and the occurrence of ADRs in hospitalized HIV/AIDS patients, nor have studies examined factors influencing MRCI values in this population. This study employs MRCI to analyze the complexity of pharmacotherapy and its determinants in HIV/AIDS patients, while evaluating whether MRCI is correlated with ADRs during hospitalization among Chinese HIV/AIDS patients.

## Methods

### Study design and population

This study constitutes a retrospective investigation. The study cohort comprised 1,010 HIV/AIDS patients discharged from Changsha First Hospital between 1 January 2022 and 30 June 2022. All confirmed cases met the diagnostic criteria outlined in the Chinese Guidelines for HIV/AIDS Diagnosis and Treatment (Acquired Immunodeficiency Syndrome Professional Group et al., 2024). Generally, a diagnosis can be made when both the initial screening test for HIV antibodies and subsequent supplementary tests yield positive results. This study was approved by the institutional Ethics Committee of the First Hospital of Changsha (Ethics Approval Number: KX-2020048) and was conducted in accordance with the Declaration of Helsinki. All enrolled participants provided written informed consent.

All data have undergone encryption and anonymization processes to safeguard the privacy of participants. Upon completion of data analysis, all records were permanently deleted to ensure additional protection of subjects’ privacy.

### Data collection and ADR assessment

The collected data encompassed the gender, age, medication allergy history, number of prescribed drugs, underlying diseases, complications, and the administration of antituberculosis medications during hospitalization among HIV/AIDS patients. All suspected ADRs in this study were retrieved from medical records. All suspected ADRs underwent causality assessment using the internationally recognized WHO-Uppsala Monitoring Centre (WHO-UMC) system (https://who-umc.org). Two clinical pharmacists and attending physicians jointly evaluated whether HIV patients had experienced ADRs based on established assessment criteria. Patients with causality assessments categorized as certain, probable, or possible were included in the study. When discrepancies arise among assessors regarding the identification or classification of adverse reactions, more authoritative experts will be consulted to make a new judgment.

### MRCI calculation

Based on the relevant litterateur (García et al., 2025), we conducted MRCI scoring for eligible patients. The assessment comprises three primary components: Part A assigns higher weights to medications with inconvenient or more challenging administration forms (e.g., oral tablets score 1 point whereas metered-dose inhalers score 4 points). Part B focuses on medications requiring more frequent dosing or stricter administration intervals, where higher scores are allocated (e.g., twice-daily regimens score 2 points, while 12-h dosing intervals score 2.5 points). Part C further evaluates treatment regimens by assessing the presence of additional instructions, with examples including “split/crush tablets” (1 point) or “taper dose as directed” (2 points). The specific content can be found in Supplementary Table S1. MRCI values were calculated separately for the day prior to admission, the day of admission, and the day of discharge.

### Data processing and statistical analysis

Statistical analysis was performed using SPSS 25.0 software. Quantitative data are presented as mean ± standard deviation (X ± s), while qualitative data are expressed as frequencies and percentages. SPSS 25.0 software analysis of the Receiver Operating Characteristic (ROC) curve. Between-group comparisons of quantitative data were conducted using t-tests, whereas chi-square tests were employed for qualitative data comparisons. Paired t-tests were utilized to analyze pre- and post-hospitalization MRCI changes. Factors with P-values <0.05 in between-group comparisons were incorporated into multivariate logistic regression analysis, with odds ratios (OR) and their 95% confidence intervals (CI) calculated. A P-value <0.05 was considered statistically significant.

## Results

### Participant characteristics and MRCI

**FIGURE 1:**
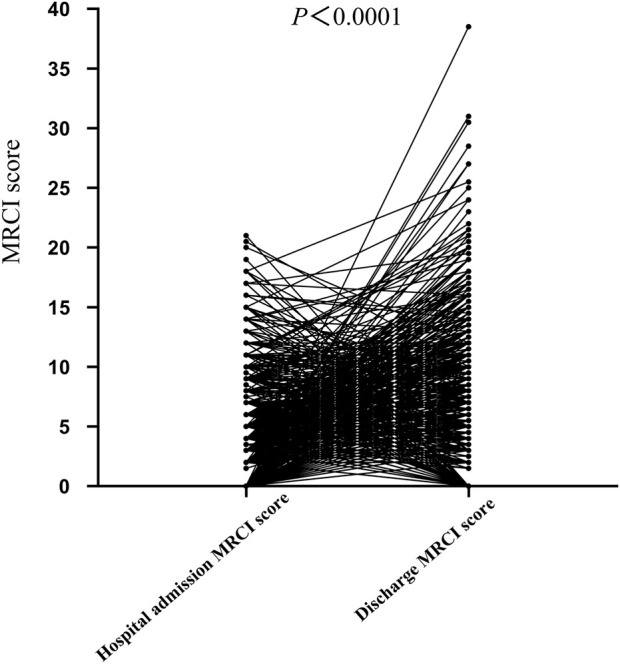
Comparison of MRCI values in HIV patients before admission and at discharge.

This study enrolled 1,010 patients with a mean age of 48.0 ± 14.7 years, comprising 831 males (82.27%) and 179 females (17.73%). Among the patients included in this study, 61.19% of the AIDS patients had comorbidities. Additionally, newly diagnosed patients, patients admitted to the hospital due to ADRs, and those with poor treatment efficacy accounted for 34.75%, 3.56%, and 0.50% respectively. During hospitalization, the average number of medication types administered was 18.69 ± 12.45. Prior to hospitalization, the patient’s MRCI was (4.09 ± 4.04), which significantly increased to (6.09 ± 6.05) at discharge, demonstrating statistical significance (*P* < 0.0001) (Figure 1).

Based on the median MRCI at admission, patients were stratified into two cohorts: the low-MRCI group (<21) and the high-MRCI group (≥21). The results demonstrated that compared with the low-MRCI group, the high-MRCI group exhibited significantly higher incidences of complications (>6 diseases), tuberculosis drugs, number of drugs used in hospital (>19 drugs), and duration of HIV infection (>0.75 years). No statistically significant differences were observed between the two groups regarding age (≥65 years), gender, history of drug allergies, or underlying comorbidities (Table 1).

**TABLE 1:** Characteristics of patient between those with Low-MRCI and High-MRCI.

Characteristic	All patients (n = 1010)	Low-MRCI (n = 491)	High-MRCI (n = 519)	P
Age (≥65 years old)	126	57	69	0.42
Male	831	405	426	0.87
History of drug allergies	18	7	11	0.41
Underlying disease	474	242	232	0.15
Complications (>6 diseases)	406	125	281	***<0.001***
Tuberculosis drugs	316	111	205	***<0.001***
Number of drugs used in hospital (>19 drugs)	496	171	325	***<0.001***
Duration of HIV infection (>0.75 years)	498	265	233	0.004

Data are n (%) or mean (+SD).

*P* value in bold italic shows that the variables are statistically significant.

### Risk factors for MRCI in AIDS patients

We conducted further logistic regression analysis to investigate the risk factors influencing patients’ MRCI. Univariate analysis revealed that complications (>6 diseases), number of drugs used in hospital (>19 drugs), and duration of HIV infection (>0.75 years) demonstrated statistically significant associations with MRCI.

Multivariate logistic regression analysis indicated that complications (>6 diseases) (OR: 2.85, *P* < 0.001), tuberculosis drugs (OR: 1.82, *P* < 0.001), and the number of drugs used in the hospital (>19 drugs) (OR: 2.02, *P* < 0.001) were independent risk factors influencing the MRCI (Table 2).

**TABLE 2:** Univariate and multivariate analysis of risk factor for MRCI values in the AIDS patients.

Variable	Low-MRCI (n = 491)	High-MRCI (n = 519)	Univariate analysis		Multi-variate analysis	
			Or (95% CI)	P	Or (95% CI)	P
Age (≥65 years old)	57	69	​	0.42	​	​
Male	405	426	​	0.87	​	​
History of drug allergies	7	11	​	0.41	​	​
Underlying disease	242	232	​	0.15	​	​
Complications (>6 diseases)	125	281	3.46 (2.65 4.51)	***<0.001***	2.85 (2.13 3.81)	***<0.001***
Tuberculosis drugs	111	205	2.24 (1.70 2.94)	***<0.001***	1.82 (1.35 2.42)	***<0.001***
Number of drugs used in hospital (>19 drugs)	171	325	3.14 (2.42 4.05)	***<0.001***	2.02 (1.52 2.68)	***<0.001***
Duration of HIV infection (>0.75 years)	265	233	1.44 (1.12 1.84)	***0.004***	​	0.069

*P* value in bold italic shows that the variables are statistically significant.

### Correlation of the MRCI with the ADRs among AIDS patients

**FIGURE 2:**
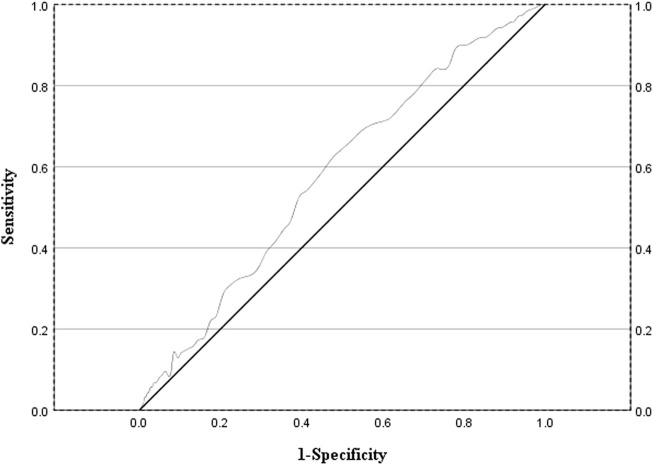
ROC curve evaluating the sensitivity and specificity of MRCI values in predicting the occurrence of adverse drug reactions in AIDS patients.

This study evaluated the discriminatory efficacy of the MRCI in distinguishing patients at risk of adverse reactions through receiver operating characteristic (ROC) curve analysis. The analysis results indicated that the area under the curve (AUC) of the MRCI for predicting adverse reactions was 0.58 (95% confidence interval: 0.54–0.62; *P* = 0.001), and its optimal cut - off value was 20.75 (Figure 2).

Among 1,010 patients, 210 experienced adverse reactions, with an incidence rate of adverse reactions at 20.79%. We classified the patients into two groups based on the occurrence of ADRs: the group with ADRs and the group without ADRs. Compared with the group without adverse reactions, the group with adverse reactions exhibited significant differences in terms of underlying diseases, complications (>6 diseases), and MRCI (>20.75) (Table 3).

**TABLE 3:** Characteristics of patient between those with ADRs and without ADRs.

Characteristic	All patients (n = 1010)	Patients with ADRs (n = 210)	Patients without ADRs (n = 800)	P
Age (≥65 years old)	126	22	104	0.33
Male	831	173	658	0.96
History of drug allergies	18	1	17	0.11
Underlying disease	474	83	391	***0.016***
Complications (>6 diseases)	406	114	292	***<0.001***
Duration of HIV infection (>0.75 years)	498	95	403	0.19
MRCI (>20.75)	519	131	388	***<0.001***

Data are n (%) or mean (+SD).

P value in bold italic shows that the variables are statistically significant.

To determine the correlation between MRCI (>20.75) and ADRs in AIDS patients, we conducted a regression analysis. Multivariate regression analysis indicated that underlying diseases (OR: 1.60, *P* = 0.004), complications (>6 diseases) (OR: 2.00, *P* < 0.001), and MRCI (>20.75) (OR: 1.42, *P* = 0.036) were independent risk factors for adverse reactions in AIDS patients (Table 4).

**TABLE 4:** Univariate and multivariate analysis of risk factor for ADRs in the AIDS patients.

Variable	Patients with ADRs (n = 210)	Patients without ADRs (n = 800)	Univariate analysis		Multi-variate analysis	
			Or (95% CI)	P	Or (95% CI)	*P*
Age (≥65 years old)	22	104	​	0.33	​	​
Male	173	658	​	0.97	​	​
History of drug allergies	1	17	​	0.14	​	​
Underlying disease	83	391	1.46 (1.07 1.99)	***0.016***	1.60 (1.16 2.20)	***0.004***
Complications (>6 diseases)	114	292	2.07 (1.52 2.81)	***<0.001***	2.00 (1.45 2.78)	***<0.001***
Duration of HIV infection (>0.75 years)	95	403	​	0.19	​	​
MRCI (>20.75)	131	388	1.76 (1.29 2.41)	***<0.001***	1.42 (1.02 1.98)	***0.036***

P value in bold italic shows that the variables are statistically significant.

## Discussion

People with AIDS typically require lifelong and complex ART regimens. Meanwhile, this population often suffers from multiple comorbidities, including cardiovascular diseases, metabolic disorders, mental disorders, and opportunistic infections. Such comorbid conditions have led to a significant increase in the use of non - antiretroviral medications (Abdelbary et al., 2023; Back and Marzolini, 2020). This study reveals that the MRCI, as a comprehensive assessment tool, is effective in quantifying medication complexity and predicting the risk of ADRs among AIDS patients. Compared with methods solely based on medication quantity statistics (such as the concept of polypharmacy), MRCI can more comprehensively and profoundly reflect the medication management challenges that AIDS patients encounter during actual drug treatment.

Research has indicated that the MRCI of AIDS patients range from 2 to 67.5 (Metz et al., 2014). A retrospective study found that the median MRCI of HIV - positive patients aged 18 and above who were receiving stable antiretroviral therapy was eight points (García et al., 2025). A multi - center cross - sectional study enrolled 74 HIV - infected individuals aged 65 years or older who were receiving antiretroviral therapy. The median complexity index of their treatment regimens was 13.0 (Gimeno-Gracia et al., 2020). Our research indicates that the average MRCI of AIDS patients during hospitalization is 20.61, which is consistent with the findings of Hirsch et al. (2014). The discrepancies in MRCI values across different studies may be attributed to the variations in the populations included. Our study solely encompasses inpatients, whereas other studies predominantly involve outpatients.

Further logistic regression analysis indicates that comorbidities, the use of anti - tuberculosis drugs, and the use of more than 19 types of in - hospital medications are independent predictors of high MRCI in AIDS patients. Among them, comorbidities or the requirement for multiple medications in anti - tuberculosis treatment is an important factor contributing to the complexity of the drug treatment regimen. In addition, the results of this study indicate that the MRCI of AIDS patients were 4.09 ± 4.04 before hospitalization and 6.09 ± 6.05 at the time of discharge (*P* < 0.0001). This is consistent with the findings of Poojar et al., which suggest that hospitalization increases the complexity of drug treatment regimens (Pooja et al., 2024).

This study also explored the relationship between MRCI and ADR. The area under the curve (AUC) of the MRCI for predicting adverse reactions is 0.58, and its optimal cut - off value is 20.75. Subsequently, we conducted a logistic regression analysis. The research results indicate that even after adjusting for potential confounding factors such as age, gender, and the number of comorbidities, MRCI >20.75 remains an independent predictor of the occurrence of ADRs. Clinicians can incorporate the calculation of patients’ MRCI values into routine drug evaluations to identify patients with an MRCI >20.75. For patients of this kind, clinicians can utilize professional drug interaction analysis tools or collaborate through multidisciplinary teams to identify potential risks of drug interactions (Conti et al., 2022; Varadarajan et al., 2025). This approach facilitates the early warning of adverse drug reactions in high - risk populations and enables targeted management based on risk stratification. Consequently, it can effectively reduce the incidence of adverse reactions and mitigate the associated risk of hospitalization.

Furthermore, in this study, most patients were HIV-infected individuals with comorbidities who likely presented with a high MRCI upon admission. For this population, clinicians should consider antiviral agents with a lower risk of drug interactions, such as integrase inhibitors, while avoiding combinations with overlapping toxicities (Tuan et al., 2024; Hidalgo-Tenorio and Martínez-Sanz, 2025; De Bellis et al., 2024). Concurrently, clinical pharmacists should perform medication reconciliation to comprehensively evaluate and optimize the therapeutic regimen, thereby contributing to the prevention of ADRs.

This study has certain limitations. Firstly, as a retrospective study, its inherent biases cannot be ignored. Secondly, the study cohort only includes patients from medical institutions in Hunan Province, resulting in limited geographical representativeness. Thirdly, there may be under - reporting of ADRs, and some cases may not be fully captured in medical records. Fourthly, this study did not conduct severity grading for the reported adverse events.

## Data Availability

The raw data supporting the conclusions of this article will be made available by the authors, without undue reservation.
