# Supplementary material for: Association of medication regimen complexity index with ADRs in HIV/AIDS patients: a retrospective cohort study
Source: Front Pharmacol. 2026 Jan 12;16:1721289. doi: 10.3389/fphar.2025.1721289 (PMC12833075; doi:10.3389/fphar.2025.1721289)
Supplement: Supplementary file 1 [file Table1.doc]

Supplement table 1 MEDICATION REGIMEN COMPLEXITY INDEX

A) Circle the weighting corresponding to each dosage form (ONCE

ONLY) present in the regimen

| Dosage Forms | | Weighting |
| --- | --- | --- |
| ORAL | Capsules/Tablets | 1 |
|  | Gargles/Mouthwashes | 2 |
|  | Gums/Lozenges | 2 |
|  | Liquids | 2 |
|  | Powders/Granules | 2 |
|  | Sublingual sprays/tabs | 2 |
| TOPICAL | Creams/Gels/Ointments | 2 |
|  | Dressings | 3 |
|  | Paints/Solutions | 2 |
|  | Pastes | 3 |
|  | Patches | 2 |
|  | Sprays | 1 |
| EAR,EYE& NOSE | Ear drops/creams/ointments | 3 |
|  | Eye drops | 3 |
|  | Eye gels/ointments | 3 |
|  | Nasal drops/cream/ointment | 3 |
|  | Nasal spray | 2 |
| INHALATION | Accuhalers | 3 |
|  | Aerolizers | 3 |
|  | Metered dose inhalers | 4 |
|  | Nebuliser | 5 |
|  | Oxygen/Concentrator | 3 |
|  | Turbuhalers | 3 |
|  | Other DPIs | 3 |
| OTHERS | Dialysate | 5 |
|  | Enemas | 2 |
|  | injections: Prefilled  Ampoules/Vials | 3 4 |
|  | Pessaries | 3 |
|  | Patient controlled analgesia | 2 |
|  | Suppositories | 2 |
|  | Vaginal creams | 2 |
| Total for Section A | |  |

B) For each medication in the regimen tick a box [√] corresponding to the dosing frequency. Then, add the no.of[√]in each category and multiply by the assigned weighting. In cases where there is no exact option, choose the best option.

| **Dosing** **Frequency** | **Medications** | | | | | | | | | | | | | | Total | Weighting | Weighting×  No.of  medications |
| --- | --- | --- | --- | --- | --- | --- | --- | --- | --- | --- | --- | --- | --- | --- | --- | --- | --- |
| Once daily |  |  |  |  |  |  |  |  |  |  |  |  |  |  |  | 1 |  |
| Once daily prn |  |  |  |  |  |  |  |  |  |  |  |  |  |  |  | 0.5 |  |
| Twice daily |  |  |  |  |  |  |  |  |  |  |  |  |  |  |  | 2 |  |
| Twice daily prn |  |  |  |  |  |  |  |  |  |  |  |  |  |  |  | 1 |  |
| Three times daily |  |  |  |  |  |  |  |  |  |  |  |  |  |  |  | 3 |  |
| Three times daily pn |  |  |  |  |  |  |  |  |  |  |  |  |  |  |  | 1.5 |  |
| Four times daily |  |  |  |  |  |  |  |  |  |  |  |  |  |  |  | 4 |  |
| Four times daily pn |  |  |  |  |  |  |  |  |  |  |  |  |  |  |  | 2 |  |
| q12h |  |  |  |  |  |  |  |  |  |  |  |  |  |  |  | 2.5 |  |
| q 12h pm |  |  |  |  |  |  |  |  |  |  |  |  |  |  |  | 1.5 |  |
| q8h |  |  |  |  |  |  |  |  |  |  |  |  |  |  |  | 3.5 |  |
| q 8h prn |  |  |  |  |  |  |  |  |  |  |  |  |  |  |  | 2 |  |
| q 6h |  |  |  |  |  |  |  |  |  |  |  |  |  |  |  | 4.5 |  |
| q6h prn |  |  |  |  |  |  |  |  |  |  |  |  |  |  |  | 2.5 |  |
| q4h |  |  |  |  |  |  |  |  |  |  |  |  |  |  |  | 6.5 |  |
| q4h prn |  |  |  |  |  |  |  |  |  |  |  |  |  |  |  | 3.5 |  |
| q2h |  |  |  |  |  |  |  |  |  |  |  |  |  |  |  | 12.5 |  |
| q2h prn |  |  |  |  |  |  |  |  |  |  |  |  |  |  |  | 6.5 |  |
| prm/sos |  |  |  |  |  |  |  |  |  |  |  |  |  |  |  | 0.5 |  |
| On alternate days or less frequently |  |  |  |  |  |  |  |  |  |  |  |  |  |  |  | 2 |  |
| Oxygen prn |  |  |  |  |  |  |  |  |  |  |  |  |  |  |  | 1 |  |
| Oxygen<15hrs |  |  |  |  |  |  |  |  |  |  |  |  |  |  |  | 2 |  |
| Oxygen>15hrs |  |  |  |  |  |  |  |  |  |  |  |  |  |  |  | 3 |  |
| **Totalfor** **Section** **B** | | | | | | | | | | | | | | | | |  |

C)Tick a box [√] corresponding to the additional directions, if present in the regimen. Then, add the no.of [√]in each category and multiply by the assigned weighting.

| **Additional** **Directions** | **Medications** | | | | | | | | total | Weighting | Weighting x | No.of | medications |
| --- | --- | --- | --- | --- | --- | --- | --- | --- | --- | --- | --- | --- | --- |
| Break or crush tablet |  |  |  |  |  |  |  |  |  | 1 |  | | |
| Dissolve tablet/powder |  |  |  |  |  |  |  |  |  | 1 |  | | |
| Multiple units at one time |  |  |  |  |  |  |  |  |  | 1 |  | | |
| Variable dose (e.g.1-2caps, 2-3 puffs) |  |  |  |  |  |  |  |  |  | 1 |  | | |
| Take/use at specified time/s (e.g.mane,nocte,8 AM) |  |  |  |  |  |  |  |  |  | 1 |  | | |
| Relation to food (e.g.pc,ac, with food) |  |  |  |  |  |  |  |  |  | 1 |  | | |
| Take with specific fluid |  |  |  |  |  |  |  |  |  | 1 |  | | |
| Take/use as directed |  |  |  |  |  |  |  |  |  | 2 |  | | |
| Tapering/increasing dose |  |  |  |  |  |  |  |  |  | 2 |  | | |
| Alternating dose(e.g.one mane&two nocte,one/two  on alternate days) |  |  |  |  |  |  |  |  |  | 2 |  | | |
| **Total** **for** **Section** **C** | | | | | | | | | | |  | | |

1.MRCI applies only to prescribed medications. All entries are to be made only based on information on the label or drug chart (at the time of dispensing or discharge). No assumptions are to be made based on clinical judgement.

2.There are three sections in the scale. Complete each section before proceeding to the next. At the end, add the scores for the three sections to give the MRCI.

3.If the same medication (same brand and same dosage form) is present more than once in different strengths in a regimen (e.g.Marevan 5mg,3mg and 1 mg mdu), it is still considered as one medication.

4.In cases where the dosage is optional, choose the dosing instruction with the smallest dose/frequency. (e.g.Ventolin MDI 1-2 puffs,2-3times daily will get weightings for metered dose inhalers, variable dose and twice daily; but not for 'multiple units at one time)

5.In certain cases the dosing frequency needs to be calculated (e.g. Ranitidine 1 mane and 1 nocte is 1 twice daily)"

6.It is possible that with certain ‘use as directed' instructions, the regimen will not get a score under dosing frequency (e.g. Prednisolone 5mg mdu)"

7.If there is more than one dosing frequency direction, they should be scored for all the dosing frequency directions (e.g.Ventolin MDI2 puffs bd and prn,will get scores for 'metered dose inhalers', multiple units at one time','" "twice daily' as well as""pm')

8.Instances where two or more medications are mutually exclusive, they need to be scored twice or more as pm with the recommended dosing frequency (e.g.Ventolin MDI or Ventolin nebuliser twice daily will get scores for both'metered dose inhalers'and 'nebuliser' under dosage forms, but needs to be scored two times for 'twice daily pm')"

9.In cases where there is no matching option, choose the closest option (e.g. six times daily could be considered as⁴q4h"")"
